# Epigenetic drug 5-azacytidine impairs proliferation of rat limb buds in an organotypic model-system *in vitro*

**DOI:** 10.3325/cmj.2013.54.489

**Published:** 2013-10

**Authors:** Vedrana Mužić, Ana Katušić Bojanac, Gordana Jurić-Lekić, Marta Himelreich, Katarina Tupek, Ljiljana Šerman, Nina Marn, Nino Sinčić, Maja Vlahović, Floriana Bulić-Jakuš

**Affiliations:** 1 Department of Rehabilitation and Orthopaedic Devices, University Hospital Centre Zagreb, Zagreb, Croatia; 2 Department of Medical Biology, University of Zagreb School of Medicine, Zagreb, Croatia; 3 Department of Histology and Embryology, University of Zagreb, School of Medicine, Zagreb, Croatia

## Abstract

**Aim:**

To establish an organotypic *in vitro* model of limb bud development to verify whether epigenetic drug and teratogen 5-azacytidine (5azaC) has an effect on limb buds independent of its effects on the placenta.

**Methods:**

Fischer strain rat fore- and hindlimb buds were microsurgically isolated from 13 days old embryos and cultivated *in vitro* for two weeks at the air-liquid interface in Eagle's minimum essential medium (MEM) with 50% rat serum. 30 µmol of 5azaC was added to the fresh medium. Overall growth was measured by an ocular micrometer. Routine histology, immunohistochemical detection of the proliferating cell nuclear antigen (PCNA), and stereological quantification of PCNA expression were performed.

**Results:**

At four time points, significantly lower overall growth was detected for fore- and hindlimb bud explants cultivated with 5azaC in comparison to controls. After the culture period, numerical density of the PCNA signal for both types of limb buds was lower than for controls (*P* < 0.001). Limb buds were initially covered by immature epithelium and contained mesenchyme, myotubes, single hemangioblasts, hemangioblast aggregates, blood islands, and capillaries. Regardless of the treatment, cartilage and epidermis differentiated, but cells and structures typical for vasculogenesis disappeared.

**Conclusion:**

Our findings, obtained outside of the maternal organism, stress the importance of compromised cell proliferation for 5azaC impact on limb buds. This investigation points to the necessity to establish alternatives to *in vivo* research on animals using teratogenic agents.

Epigenetics deals with heritable changes in gene expression or cellular phenotype. They are caused mainly by changes in DNA methylation and histone code without changes in the underlying DNA sequence. Epigenetic research has especially been focused on development (1) and cancer (2,3). This research led to an entirely new class of so-called epigenetic drugs, which have recently entered clinical trials (4). Possible side-effects and teratogenicity caused by these drugs are of substantial importance for human medicine.

One of the archetypal epigenetic therapeutic agents, the DNA demethylating agent 5-azacytidine (5azaC) has been approved by US Food and Drug Administration for treatment of myelodysplastic syndrome in all its subtypes (5). Although the rationale for its approval was its ability to demethylate and activate genes such as tumor suppressors, only recently its genome-wide activity has been addressed (6) and it has been found out that it is also able to reorganize histone modification patterns (7). Because it changes gene expression necessary for the normal course of development, 5azaC has been known to influence developmental parameters such as survival, differentiation, growth, and morphogenesis. Applied *in vivo* during rat gestation, it was embryotoxic or caused malformations in a stage-specific manner. Until the 11th day, embryos were susceptible to resorptions, while later overall growth (weight and crown-rump length) was impaired. The most critical period for induction of limb malformations was from the 12th to 13th day (8). Because 5azaC also impaired placental growth and morphology, the question remains whether it is directly affecting limb buds or acting indirectly by affecting placental function (9,10).

It is possible to investigate the influence of 5-azaC on the embryo in a specific *in vitro* model of rat embryonic development at the air-liquid interface, without the confounding change in the placenta (11,12). When applied in serum-free conditions to the gastrulating rat embryo-proper (consisting of ectoderm, mesoderm, and endoderm), 5azaC impaired survival, growth, and differentiation (11) but in serum-supplemented conditions it promoted differentiation of muscle (12). In a culture of a younger, pre-gastrulating embryo (consisting of epiblast and hypoblast), it promoted differentiation of muscle, cartilage, blood islands, and neural tissue (13). Recent results associate the impact of 5azaC in an *in vitro* cell culture developmental model with a decrease in cell proliferation (14). On the other hand, in a cartilaginous organ transplanted *in vivo* it enhanced cell proliferation (15). This may seem to be controversial but 5azaC acted specifically for each developmental model system.

According to the original organ-culture model at the air-liquid interface established before for investigation of developmental processes in the rat embryo (12), we aimed to establish a new *in vitro* organotypic model-system for rat limb bud development. In this model, the overall growth of explanted limb buds could be assessed at several points during the culture period and, at the end of culture, the ability for cell proliferation at the single cell level could be assessed by a cell proliferation marker. The proliferating cell nuclear antigen (PCNA) expression was stereologically quantified similarly as in our *in vivo* experiments (10). Establishing such an *in vitro* experimental model, in which the influence of 5azaC on maternal organism is avoided, a clear answer could be obtained about the susceptibility of limb buds to 5azaC. Moreover, it would be possible to resolve the dilemma about 5azaC impact on cell proliferation in the developing limb bud as a whole organ.

## Material and methods

### Isolation of rat limb buds

Fischer strain rats were mated overnight and the finding of sperm in the vaginal smear next morning designated the day 0 of pregnancy. Females were euthanized with anesthetic and 13 days old fetuses were isolated. A total of 128 fore- and hindlimb buds were microsurgically isolated under the dissecting microscope by Graeffe’s knife and a watchmaker’s forceps and rinsed in phosphate buffered saline (PBS). The experiments were approved by the Ethics Committee of University of Zagreb School of Medicine.

### Organ culture *in vitro*

Isolated limb buds (N = 32 per group) were grown in disposable organ culture dishes with a central well (Falcon No 3037, Becton Dickinson, Oxford, UK) on stainless steel grids made at the Department of Medical Biology, University of Zagreb School of Medicine, and covered with a piece of lens paper (Whatman Grade 105, Sigma Aldrich, Taufkirchen, Germany). Eagle's minimal essential medium (MEM) with Hanks’ salt solution (Sigma Aldrich) with 50% rat serum was poured under the grid to wet the paper. Rat serum was obtained from aortal bifurcation of anesthetized male rats and immediately inactivated at 56°C. 30 µM 5azaC (Sigma Aldrich) in MEM and 50% rat serum was used for cultivation of experimental samples. Limb buds were grown in an incubator at the air-liquid interface for two weeks in 5% C0_2_ and 95% air at 37°C. The culture medium was changed five times during the culture period.

### Overall growth of explants

To monitor growth, ellipsoid explants major and minor diameters were measured by an ocular micrometer at the beginning of the culture period and four times at the time of the culture medium change (16,17). Measures were included in the formula for ellipse area (A = π × major diameter × minor diameter / 4). Calculated A values (ranging from 2.4 to 2.6 mm^2^) were normalized by division to values of the initial measurement and used as the measure of overall growth (A/A_0_)_._ Therefore, A/A_0_ was 1 for the day 0 when the explants were plated at the air-liquid surface.

### Histology and immunohistochemistry

Explants were fixed by St. Marie solution (1% acetic acid in 96% ethanol, +4°C), embedded in paraffin and 5 μm histological sections were prepared. Some samples were processed for routine histology and stained with hematoxylin-eosin (HE) or Masson trichrome stain. For immunohistochemistry, sections (5 μm) were put on silanized slides (DAKO, Glostrup, Denmark) and air-dried for 24 hours at room temperature. Sections were deparaffinized in xylene (2 × 5 minutes), treated with absolute ethanol and 96% ethanol (2 × 5min), 70% ethanol, and PBS (5 minutes). Epitope retrieval was done in the Target Retrieval Solution (DAKO ChemMateTM) ( × 10) in a microwave oven according to the manufacturer’s instructions. Monoclonal mouse PCNA, Clone PC 10 (DAKO), was diluted to 1:100 and applied overnight. Negative controls were treated with an unspecific antibody (No. V 1617 mouse IgG_1_, DAKO). Labeled streptavidin-biotin kit (Dakocytomation, LSAB®2 System-HRP, DAKO), Streptavidin/HRP (DAKO), and chromogen system (Dakocytomation, Liquid DAB+ substrate, DAKO) was used according to manufacturer’s instructions. Sections were briefly counterstained with hematoxylin, washed with distilled water, then for 20 minutes in tap water, again for 3 minutes in distilled water, and covered with 50% glycerol in PBS.

### Stereology

Cells positive for PCNA were stained by DAB (brown). The signal was always present in the nuclei although the cytoplasmic signal together with the conspicuous nuclear staining was also found. Nuclei of the cells that were negative for PCNA were stained by the counterstain (blue) (9,10,15). Randomly selected paraffin blocks (6 for each group of explants – total 24 blocks) were used for stereological analysis of PCNA-positive cells. Five consecutive sections were taken randomly from each block and quantitative stereological analysis of numerical density (Nv) was performed by Nikon Alphaphot binocular light microscope (Nikon, Vienna, Austria) using Weibel’s multipurpose test system with 42 points (M 42) at a magnification of 400 × . The area tested (A_t_) was 0.0837 mm^2^. For each investigated group the orientation/pilot stereological measurement was carried out on 10 fields in order to define the number of fields to be tested. The results were included in the formula n = (200/y × s/ arithmetic mean)^2^ (18), where n is the number of fields to be analyzed, the arithmetic mean is of the orientation sample, s is standard deviation, and y the allowed deviation from the arithmetic mean at the 95% confidence interval, which reflects the significance level of 0.05. At least 15 fields per group were analyzed. The numerical density of PCNA-positive cells was determined by the point counting method. Numerical density (Nv) was calculated by the formula: Nv = N/A_t_ × D, where N is the number of PCNA-positive cells in the tested area. The mean tangential diameter (D) was calculated by Ellipse 3D program and for 100 cells it was 0.0147 mm and the test-area (A_t_) was 0.0837 mm^2^. Results were analyzed by *t* test and the statistical significance level was *P* < 0.05.

## Results

### Overall growth

Throughout the culture period, overall growth of explants was lower in 5azaC treated explants. For the forelimb buds, a significant difference between experimental and control groups was first discovered at the fourth day of the culture, and for the hindlimb buds a significant difference was first discovered at the sixth day (Table 1).

**Table 1:** Decrease in overall growth and the proliferating cell nuclear antigen (PCNA) expression in rat limb buds cultivated with 5azaC

Forelimb buds	Controls		5azaC		t	*P*
Overall growth	No. of samples	A/A_0_* (mean±SE^†^)	No. of samples	A/A_0_ (mean±SE)		
Day 4	19	1.194 ± 0.050	17	0.972 ± 0.050	3.112	0.004
Day 6	16	1.155 ± 0.041	17	0.882 ± 0.034	5.151	<0.001
Day 8	14	1.015 ± 0.057	17	0.795 ± 0.040	3.251	0.003
Day 11	8	1.214 ± 0.082	17	0.7559 ± 0.051	4.915	<0.001
PCNA expression	No. of samples /microscopic fields	Nv of PCNA in mm^-3^ (mean±SE)	No. of samples /microscopic fields	Nv of PCNA in mm^-3^ (mean±SE)		
Day 14	6 /15	65755 ± 1672	6 / 42	50824 ± 2325	4.770	<0.001
Hindlimb buds	No. of samples	A/A_0_ (mean±SE)	No. of samples	A/A_0_ (mean±SE)		
Day 4	18	1.066 ± 0.055	31	1.137 ± 0.040	1.055	0.297
Day 6	17	1.164 ± 0.077	19	0.864 ± 0.028	3.813	0.001
Day 8	17	1.041 ± 0.070	19	0.740 ± 0.043	3.762	0.001
Day 11	17	1.089 ± 0.075	19	0.666 ± 0.028	5.522	<0.001
PCNA expression	No. of samples/ microscopic fields	Nv of PCNA in mm^-3^ (mean±SE)	No. of samples/microscopic fields	Nv of PCNA in mm^-3^ (mean±SE)		
Day 14	6 /33	64380 ± 2124	6/ 31	45042 ± 1726	7.000	<0.001

*A/A_0_ – explant area/explant area at the beginning of culture.

†SE – standard error of the mean.

### Proliferating cell nuclear antigen expression

**Figure 1:**
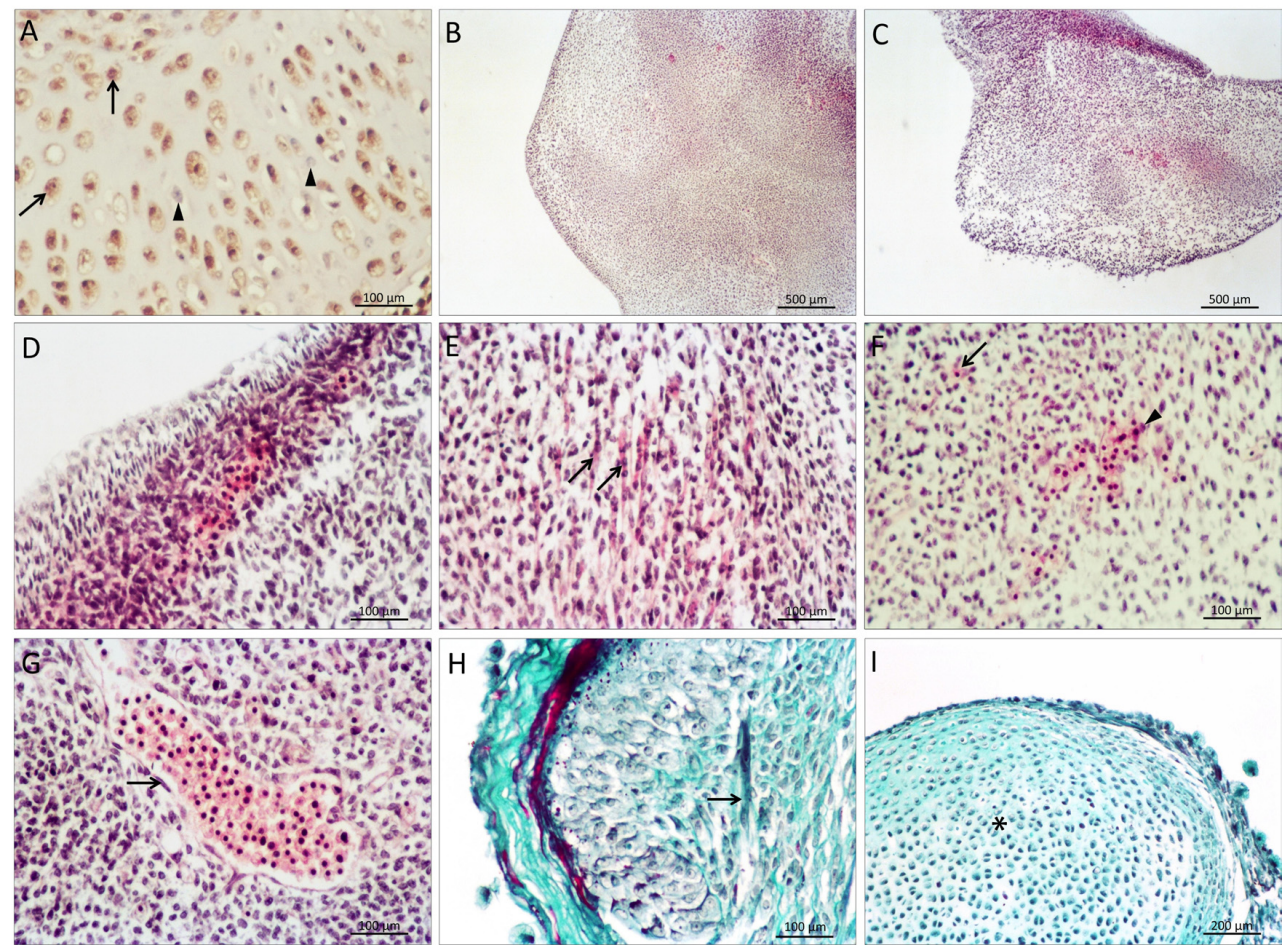
Proliferating cell nuclear antigen (PCNA) expression in single cells of the limb bud. (**A**) PCNA signal (arrow) in the forelimb bud cultivated in Eagle’s minimum essential medium (MEM) with rat serum. Internal negative control (arrowhead). DAB, contrasted with hematoxylin (HE). Histology of limb buds. Differentiation in 13 days old rat limb buds before cultivation (**B, C, D E, F, G**). (**B**) Forelimb bud, HE. (**C**) Hindlimb bud, HE. (**D**) Immature superficial epithelium in a hindlimb bud, HE. (**E**) Myotubes in a forelimb bud (arrows), HE. (**F**) Single hemangioblasts (arrow) and their aggregates (arrowhead) in a hindlimb bud, HE. (**G**) Capillary in a hindlimb bud (arrow), HE. Differentiation of rat hindlimb buds cultivated in MEM with serum for two weeks (**H, I**). (**H**) Limb bud covered by keratinized stratified epithelium. A myotube in the mesenchyme (arrow), Masson. (**I**) Limb bud with differentiated cartilage (asterisk), Masson.

The expression of the proliferating cell nuclear antigen, a marker of proliferating cells, was found in all explants regardless of the culture conditions but not in all cells (Figure 1A). Numerical density (Nv) of PCNA signals was significantly lower for explants treated with 5azaC (Table 1).

### Differentiation

Native 13 days old limb buds explanted from the rat embryo contained a central mass of mesenchyme covered with immature epithelium (Figure 1B, C, D). Within the mesenchyme myotubes (Figure 1E) and various stages of vasculogenesis, such as single hemangioblasts, their aggregates, blood islands and capillaries were found (Figure 1F, G).

Explants survived for two weeks *in vitro* at the air-liquid interface. In those limb buds, keratinized epidermis differentiated and it contained all five layers, among which there was a prominent *stratum granulosum,* typical for the thick skin (Figure 1H). Cartilage enveloped in perichondrium differentiated (Figure 1I). Peripheral areas typical for apposition growth and centrally positioned areas typical for interstitial growth were present within the cartilage. Interstitial growth was recognized by the presence of several chondrocytes in a single lacuna (isogenic group). The lacunas were surrounded by the extracellular matrix. While myotubes were still occasionally found, cells and structures typical for vasculogenesis were absent from the explants after the cultivation period *in vitro* (Figure 1H, I). Careful histological examination revealed no difference regarding differentiation between explants grown in control conditions and those grown in experimental conditions with 5azaC.

## Discussion

By using a novel approach in which limb buds were subjected to 5azaC outside of the maternal organism, we analyzed its impact on the limb bud development. For the first time it was shown that it impaired both overall growth and ability for cell proliferation in the whole developing limb bud. In such a system, tissue interactions needed for the limb bud development are partially preserved, which makes this system more similar to *in vivo* situation than simple cell cultures and therefore more suitable as an alternative to *in vivo* research (11).

Negative influence upon overall growth took place earlier for the forelimbs than for the hindlimbs, possibly because mammalian fore limbs develop earlier than hind limbs (19). The marker of cell proliferation which was used (PCNA) is a ring protein that functions as a sliding clamp necessary as an essential cofactor for DNA synthesis (20). Although its immunohistochemistry levels in rat renewing tissues were found to be higher than those of other cell proliferation markers, those markers sometimes could not be visualized due to technical problems (21). In our current experiments, in the same way as in previously published studies (9,10,15), PCNA signal was always easily detected. The quantitative difference in its expression (Nv), found between experimental and control groups of limb buds, was in accordance with the results obtained by measurements of the overall explant growth.

Because vertebrate limb development is first characterized with a remarkably rapid proximo-distal limb bud outgrowth (22), we may suppose that malformations of limbs found as a result of 5azaC application during gestation are predominantly caused by its antiproliferative activity such as shown in our study. Interestingly, the rationale underlying the low dose therapy with the DNA demethylating agent currently approved for myelodysplastic syndrome is based on the theory that induction of hypomethylation with subsequent re-expression of methylated genes will induce gene expression patterns and beneficially result in differentiation, apoptosis, or senescence of the malignant clone (23). In addition, a decrease in cell growth with an arrest of the cell cycle at G0/G1 phase seems to precede such events (24).

In our system, differentiation of isolated limb buds proceeded. By carefully performed light microscopy, advanced differentiation of cartilage and epidermis was discovered after a two-week culture with no difference found between explants treated or untreated with 5azaC. However, during *in vitro* culture, vasculogenesis found in 13 days old isolated limb buds seems to have disappeared. The reason for this was probably the lack of specific *in vivo* signals, which are absent from the simple *in vitro* environment (15).

The *in vitro* rat developmental model used in this investigation is in accordance with the need to find alternatives to *in vivo* developmental toxicity studies because of ethical and economical constrains. This includes search for novel *in vitro* biomarkers. Although two rat assays have already been validated by the European Centre for the Validation of Alternative Methods, they deal with limb buds either dissociated (micromass test) or developed after two days in the whole rat embryos (WEC) isolated at GD10. In the micromass test, a strong embryotoxic compound was classified as nonembryotoxic and WEC was found to be predictive only in combination with a 3T3 cell assay (25-27). These models are different from our organotypic model, which enables investigation of substance impact for the prolonged period of two weeks in an organ that is achieving a high degree of differentiation, higher than in WEC. Therefore, our rat limb bud model-system with quantitative endpoints defined in this investigation (overall growth and cell proliferation) might be proposed as a complementary model, which may provide more information about developmental toxicity during organogenesis.

To conclude, by the novel *in vitro* approach we eliminated maternal and placental effect and gave a more straightforward answer about the negative impact of 5azaC on limb bud development. The results of this investigation stress the impact of decreased proliferation for 5azaC teratogenicity.
